# Association Between Ketogenic Diet and Overactive Bladder: The Mediating Roles of Dietary Inflammatory Index and Weight‐Adjusted Waist Index

**DOI:** 10.1002/fsn3.71587

**Published:** 2026-02-24

**Authors:** Xuefeng Jin, Tong Zhang, Hao Li, Jie Wang, Shiquan Xu, Jingping Ge, Zizhi Li, Xiangrui Kong, Junlin Chen, Xuejiao Wen, Wenhui Tong, Xiaoyan Liu, Hangxu Li

**Affiliations:** ^1^ Department of Urology, the Third Affiliated Hospital of Jinzhou Medical University Jinzhou Medical University Jinzhou Liaoning China; ^2^ Organ Transplantation Clinical Medical Centre of Xiamen University, Department of General Surgery, Xiang'an Hospital of Xiamen University, School of Medicine Xiamen University Xiamen China; ^3^ Organ Transplantation Institute of Xiamen University, Xiamen Human Organ Transplantation Quality Control Centre, Xiamen Key Laboratory of Regeneration Medicine, School of Medicine Xiamen University Xiamen China; ^4^ Department of Urology, Affiliated Taikang Xianlin Drum Tower Hospital Medical School of Nanjing University Nanjing China; ^5^ Department of Urology Clinical College of Wuhan University Nanjing Jiangsu China; ^6^ Department of Clinical Medicine Medical School of Nanjing University Nanjing Jiangsu China; ^7^ Beijing Fengtai No. 10 Cadre Sanatorium of the Beijing Garrison District Beijing China

**Keywords:** dietary inflammatory index, ketogenic diet, NHANES, overactive bladder, weight‐adjusted‐waist

## Abstract

Overactive bladder (OAB) is characterized by urinary urgency, often accompanied by increased frequency and nocturia, and is associated with impaired quality of life and substantial healthcare burden. Although ketogenic dietary patterns have been linked to weight reduction, decreased systemic inflammation, and improved metabolic profiles, their relationship with OAB remains unclear. This study aimed to examine the association between the dietary ketogenic ratio (DKR) and OAB, and to explore the potential mediating roles of the Weight‐adjusted‐waist index (WWI) and the Dietary Inflammatory Index (DII). Data were derived from 23,763 participants in the 2009–2018 National Health and Nutrition Examination Survey (NHANES), including 4991 individuals reporting OAB. Dietary intake was assessed using two 24‐h dietary recalls. DKR, DII, and WWI were calculated using validated approaches. Multivariable logistic regression, subgroup analyses, and smoothed curve fitting were performed to evaluate the association between DKR and OAB, and mediation analyses were conducted to assess indirect effects. Each unit increase in DII was associated with 11% higher odds of OAB (OR = 1.11, 95% CI: 1.06–1.16), whereas each unit increase in DKR was associated with 43% lower odds of OAB (OR = 0.57, 95% CI: 0.42–0.77). Compared with the lowest quartile, participants in the highest quartile of DKR had a significantly lower prevalence of OAB. Mediation analyses suggested that DII and WWI accounted for 8.29% and 6.57% of the association between DKR and OAB, respectively. Higher DKR was associated with a lower prevalence of OAB, and this relationship was partially mediated by dietary inflammatory potential and central adiposity. These findings highlight the potential relevance of dietary patterns in OAB prevention and warrant further prospective investigation.

## Graphical abstract

Higher dietary ketogenic ratio was associated with lower prevalence of overactive bladder, partially mediated by dietary inflammation and central adiposity in U.S. adults (NHANES 2009–2018).

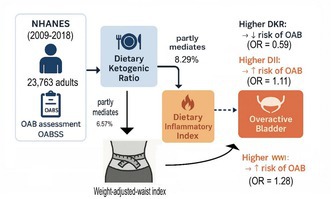

## 1 Introduction

Overactive bladder (OAB), as defined by the International Continence Society (ICS), is a symptom complex characterized primarily by urinary urgency, usually accompanied by increased frequency and nocturia, with or without urgency urinary incontinence (UUI), in the absence of urinary tract infection or other obvious local pathologies affecting the bladder or urethra (Irwin et al. 2006). OAB is highly prevalent and impairs quality of life, affecting daily activities such as work, travel, exercise, sexual activity, and sleep in both men and women (Staskin et al. 2020; Robinson et al. 2025). In recent years, the prevalence of OAB has been rising, potentially due to increasing psychosocial stress and lifestyle changes (Al Edwan et al. 2021). OAB not only poses considerable physical and psychological burdens on individuals but also contributes to substantial healthcare costs (Sexton et al. 2011). Given the aging global population and the age‐related increase in OAB prevalence, its economic impact is expected to grow (Chow et al. 2018). In spite of its high predominance and societal burden, the underlying etiology of OAB remains unclear, highlighting the importance of identifying modifiable risk factors for timely intervention (Chu and Dmochowski 2006).

The Ketogenic diet (KD), a high‐fat, low‐carbohydrate dietary pattern, has been used for nearly a century as a therapeutic strategy for epilepsy and has recently gained popularity for weight loss and metabolic health improvement in obese individuals (Deng et al. 2025; Mohammadifard et al. 2022). KD shifts energy metabolism from carbohydrates to fats, causing ketone molecules like acetoacetate and β‐hydroxybutyrate to be produced (Zhu et al. 2022). Numerous health advantages are thought to result from this metabolic shift, such as increased insulin sensitivity, decreased systemic inflammation, and improved cognitive performance (Paoli et al. 2021; Castro et al. 2024; Rong et al. 2024; Nagpal et al. 2019).

To objectively assess the extent to which a diet induces nutritional ketosis, the dietary ketogenic ratio (DKR) has been developed, which is calculated based on the ratio of macronutrients with ketogenic versus anti‐ketogenic properties. Another dietary measure, the Dietary Inflammatory Index (DII), quantifies the inflammatory potential of an individual's diet by analyzing the effects of various foods and nutrients on the inflammatory response (Wang et al. 2024; Chang et al. 2024). Elevated DII scores have been associated with increased risk of multiple chronic diseases (Xie et al. 2023). Notably, one recent study found that a higher weight‐adjusted waist index (WWI) was positively associated with increased OAB risk, with DII acting as a potential mediator, indicating that eating a diet low in inflammation may help avoid OAB (Ishizuka et al. 2024).

Given that DKR reflects a high‐fat, low‐carbohydrate dietary structure and DII captures the inflammatory nature of dietary intake, both indices may influence OAB risk by modulating systemic inflammation. However, to the best of our knowledge, no study has yet explored the relationship between ketogenic dietary patterns and OAB. Therefore, this research aimed to investigate the association between DKR and OAB using information from the National Health and Nutrition Examination Survey (NHANES), and to investigate further the possible mediation function of DII and WWI in this relationship. The findings may offer novel insights into dietary strategies for OAB management and prevention.

## 2 Methods

### 2.1 Study Participants

NHANES is a landmark, nationally representative examination program launched in the 1960s and remains a cornerstone of the U.S. public health surveillance system. Performed by the Centres for Disease Control and Prevention's (CDC) National Centre for Health Statistics (NCHS), NHANES transitioned to a continuous survey design in 1999, with data collected biennially (Jin, Tong, et al. 2024). The program employs a multi‐stage, stratified sampling strategy and includes detailed questionnaires, standardized physical examinations, and comprehensive laboratory assessments. These components yield rich data on demographic characteristics, dietary intake, anthropometric measures, and biochemical markers, enabling a holistic understanding of the health and nutritional status of the U.S. population. All NHANES protocols are approved by an institutional review board, and informed consent is obtained from all participants (Li et al. 2025). The de‐identified datasets are publicly available, ensuring transparency and accessibility for researchers and policymakers. NHANES serves as a critical resource for health research and evidence‐based public health decision‐making.

**FIGURE 1:**
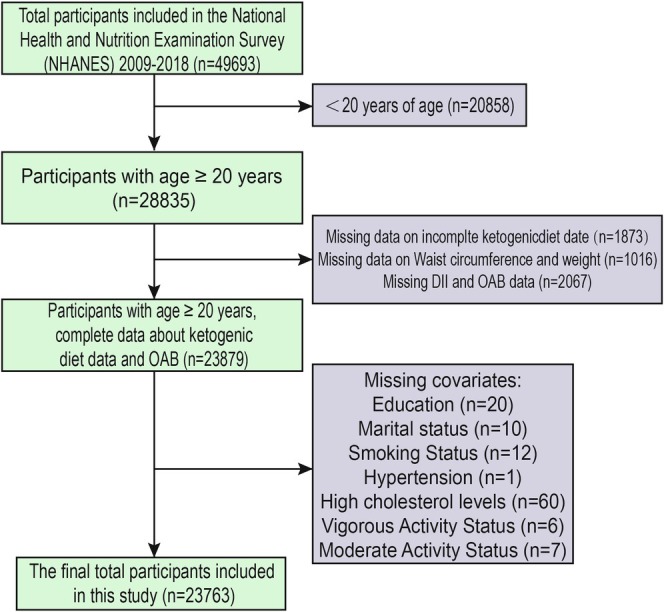
Research participants screening flowchart.

A systematic and rigorous sample selection protocol was employed in this study to ensure data completeness, representativeness, and analytical validity. As shown in Figure 1, data from seven NHANES cycles (2009–2018) were utilized, encompassing an initial pool of 49,693 participants. The exclusion process proceeded in three main steps: (1) individuals under 20 years of age were excluded (*n* = 20,858), leaving 28,835 participants; (2) participants missing ketogenic dietary data were excluded (*n* = 1873), missing waist circumference and weight data were excluded (*n* = 1016); and (3) individuals with incomplete information on DII or OAB were excluded (*n* = 2067), as well as other covariates (total *n* = 116). The final analytic sample consisted of 23,763 participants. This tiered selection process helped ensure internal consistency and minimized potential confounding, thereby enhancing the credibility and generalizability of the findings.

### 2.2 OAB Assessment

In clinical practice, accurate identification and diagnosis of overactive bladder (OAB) rely on standardized symptom assessment (Homma and Gotoh 2009). Based on its definition, urgency urinary incontinence, and nocturia are key features of OAB. In this study, OAB was assessed using three NHANES questionnaire items (KIQ044, KIQ450, and KIQ480). To quantify symptom severity, the Overactive Bladder Symptom Score (OABSS) was employed, with scoring details provided in Table S1. Scores from the urgency incontinence and nocturia components were added together to determine the total OABSS. A score of ≥ 3 was defined as indicative of OAB (Sillén et al. 2020; Gao et al. 2024).

### 2.3 Measurement of DKR

To assess dietary patterns capable of inducing nutritional ketosis, the dietary ketogenic ratio (DKR) was employed as a quantitative index. This ratio was calculated based on the relative proportions of the three principal macronutrients—fat, protein, and net carbohydrates—each of which exerts distinct ketogenic or anti‐ketogenic properties. Specifically, the DKR was derived using a formula originally proposed by Withrow (1980), where greater weights are assigned to the ketogenic potential of fat and protein, while carbohydrates are incorporated as anti‐ketogenic contributors. The mathematical expression is as follows:

$$\mathrm{DKR}=\frac{0.9\times \mathrm{fat}\left[g\right]+0.46\times \text{protein}\left[g\right]}{0.1\times \mathrm{fat}\left[g\right]+0.58\times \text{protein}\left[g\right]+\mathrm{net}\;\text{carbohydrates}\left[g\right]}$$

A higher DKR reflects a dietary macronutrient composition characterized by a higher relative fat content and lower carbohydrate availability, which is more conducive to ketone body production (Liu et al. 2025). In this population‐based study, DKR was quantified based on its weighted distribution and analyzed as a continuous variable and in quantiles, with higher values indicating a greater ketogenic tendency rather than a clinical definition of nutritional ketosis. This index has been widely applied in nutritional epidemiology and clinical studies to provide an objective measure of the ketogenic quality of dietary intake, facilitating the comparison of different dietary patterns in relation to metabolic outcomes.

### 2.4 Definition of DII

Two 24‐h dietary recall interviews conducted by qualified staff as part of NHANES were used to collect dietary consumption data. The first interview was conducted in person, followed by a second interview via telephone 3–10 days later. Respondents reported all foods and beverages consumed in the previous 24 h. The average of the two recalls was used to estimate the daily intake (Jin, Tong, et al. 2024; Jin et al. 2025). Nutrient content and energy intake were calculated using validated food composition databases (Jin, Sun, et al. 2024). The conventional method outlined by Shivappa et al. was used to compute the Dietary Inflammatory Index (DII) (Shivappa et al. 2014). It is a literature‐derived tool designed to quantify the inflammatory potential of a diet based on 45 dietary components. Although full data on all components may not always be available, the DII can still be computed using partial data following the original standardized methodology. In the present study, the specific dietary components included and excluded, as well as a worked example illustrating the step‐by‐step calculation of the partial DII, are provided in Supporting Information: Text 1. For each nutrient or food component iii, the individual intake amount (*Xi*) was first standardized using the global reference mean (*Xi*) and standard deviation (SDi) to obtain a *Z*‐score. The *Z*‐score was then converted into a percentile, centered (multiplied by 2 and subtracted by 1), and subsequently multiplied by the corresponding inflammatory effect score (*βi*) derived from the literature. The final DII score for each participant was obtained by summing the weighted values across all components:

$$\mathrm{DII}=\sum_{i=1}^{n}\left(\left(\frac{\mathrm{Xi}-\text{mean}\left(\mathrm{Xi}\right)}{\mathrm{SDi}}\right)\times 2-1\right)\times \mathrm{\beta i}$$

### 2.5 Assessment of Weight‐Adjusted‐Waist Index

An indicator of body measurements used to evaluate central obesity is the weight‐adjusted waist index, which is based on weight and waist circumference. Trained health technicians from NHANES provided body measuring information on weight and WC. Weight in kilograms divided by weight in centimeters was used to calculate each participant's WWI, where weight is in kilograms and WC is in centimeters (Park et al. 2018). Increased adiposity is indicated by higher WWI readings. The mathematical expression is as follows:

$$\mathrm{WWI}=\frac{\;\text{waist circumference}\left(\mathrm{cm}\right)}{\sqrt{\text{weight}\left(\mathrm{kg}\right)}}$$

### 2.6 Covariables

Based on prior research, we selected potential confounders that may affect OAB, including age, gender, education, race, PIR, marital status, total cholesterol levels, hypertension, alcohol use, smoking, diabetes, and status of exercise (Ikeda et al. 2011; McKellar et al. 2019).

Age and PIR were treated as continuous variables. Gender, race, education, and marital status were considered as categorical variables. If a participant answered “yes” to smoking, they were classified as smokers (SMQ020). Drinking alcohol was determined using the drinking question (ALQ130), classifying individuals as non‐drinkers if they consumed fewer than 12 drinks per year and as drinkers if they consumed 12 or more. Detailed specifications for SMQ020 and ALQ130 are provided in the Supporting Information: Document.

Individuals were classified as having hypertension if their average systolic blood pressure from three readings was ≥ 140 mmHg, their diastolic blood pressure was ≥ 90 mmHg, they were taking antihypertensive medication, or they answered “yes” to being diagnosed with hypertension. Diabetes was identified based on a “yes” response to a diabetes diagnosis, the use of glucose‐lowering medication or insulin, or meeting diagnostic thresholds for glycosylated hemoglobin (≥ 6.5%) and fasting blood glucose (≥ 126 mg/dL). There were two categories for total cholesterol levels: low (< 240 mg/dL) and high (≥ 240 mg/dL). Previously told by a doctor that they had hypercholesterolemia or were on lipid‐lowering medication, they were considered to have a hypercholesterolemic state; otherwise, they were considered not to be associated with hypercholesterolemia.

### 2.7 Statistical Analysis

All analyses were conducted using NHANES‐recommended sampling weights to ensure national representativeness (Li et al. 2025; Jin, Tong, et al. 2024; Jin et al. 2025; Pulavarty et al. 2022). The 2‐day dietary recall weight was employed and adjusted for the study period (2009–2018) by multiplying by 1/5. Categorical variables were summarized as frequencies and percentages, and continuous variables as means ± standard errors (SE). Group differences between participants with and without OAB were evaluated using weighted *t*‐tests for continuous variables and Chi‐squared tests for categorical variables.

Weighted multivariable logistic regression models were used to assess associations between DKR and OAB, while weighted linear regression was employed to assess the connection between DKR, WWI, and DII. Three hierarchical models were built: Model 1: unmodified; Model 2: adjusted for age, gender, race/ethnicity, marital status, education, and PIR; Model 3: additionally adjusted for smoking, alcohol use, hypertension, diabetes, and hyperlipidemia. Results were reported as odds ratios (OR) or regression coefficients (*β*) with 95% confidence intervals (CIs). Subgroup analyses were performed to explore potential effect modification. Restricted cubic spline (RCS) modeling was applied to assess non‐linear relationships.

A mediation analysis was carried out if both the relationship between DKR, WWI, and DII and that between WWI, DII, and OAB were statistically significant, in order to examine whether DII and WWI mediated the association between DKR and OAB. To further address sex‐specific factors, we conducted a female‐only analysis excluding pregnant women at the time of examination. Pregnancy status was identified from NHANES reproductive health data, and women who were pregnant (*n* = 227) were excluded from this subgroup analysis to minimize pregnancy‐related confounding of lower urinary tract symptoms. Weighted analyses, spline fitting, and visualizations were performed using the “survey” and “ggplot2” packages in R (version 4.5.1). Statistical significance was established for *p*‐values with two tails less than 0.05.

## 3 Result

### 3.1 Baseline Characteristics

A total of 23,763 participants were included in the analysis, with a weighted prevalence of overactive bladder (OAB) of 21%. Baseline characteristics differed significantly between participants with and without OAB (Table 1). Individuals aged ≥ 40 years and females were more likely to report OAB compared with younger participants and males (both *p* < 0.001). Significant differences were also observed across socioeconomic factors, including education level, poverty–income ratio, marital status, and race/ethnicity (all *p* < 0.001).

**TABLE 1:** Weighted baseline characteristics stratified by OAB.

Characteristic	Overall, *N* = 109,104,450 (100%)	Non‐OAB, *N* = 86,381,817 (79%)	OAB, *N* = 22,722,633 (21%)	*p*
No. of participants in the sample	23,763	18,772	4991	—
Age (%)				< 0.001
20–40	37,095,513 (34%)	35,416,545 (41%)	3,408,395 (15%)	
> 40	72,008,937 (66%)	50,965,272 (59%)	19,314,238 (85%)	
Gender (%)				< 0.001
Female	55,643,270 (51%)	41,463,272 (48%)	14,088,032 (62%)	
Male	53,461,181 (49%)	44,918,545 (52%)	8,634,601 (38%)	
Race (%)				< 0.001
Non‐Hispanic White	76,373,115 (70%)	59,603,454 (69%)	15,224,164 (67%)	
Non‐Hispanic Black	10,910,445 (10%)	8,551,800 (9.9%)	3,635,621 (16%)	
Other	12,001,490 (11%)	10,365,818 (12%)	2,499,490 (11%)	
Mexican American	9,819,401 (9%)	7,860,745 (9.1%)	1,363,358 (6%)	
Married/live with partner (%)				< 0.001
No	39,277,602 (36%)	30,233,636 (35%)	9,316,279 (41%)	
Yes	69,826,848 (64%)	56,148,181 (65%)	13,406,353 (59%)	
Education level (%)				< 0.001
Below high school	15,274,623 (14%)	11,229,636 (13%)	5,453,432 (24%)	
High School or above	93,829,819 (86%)	75,152,181 (87%)	17,269,201 (76%)	
PIR (%)				< 0.001
Poor	21,820,305 (20%)	15,548,727 (18%)	6,362,337 (28%)	
Not poor	87,283,560 (80%)	70,833,090 (82%)	16,360,296 (72%)	
Smoking (%)				< 0.001
Never	60,007,448 (55%)	49,237,636 (57%)	11,134,090 (49%)	
Former	27,276,375 (25%)	19,867,817 (23%)	7,044,016 (31%)	
Current	21,820,893 (20%)	17,276,363 (20%)	4,544,527 (20%)	
Drinking (%)				< 0.001
Never	10,910,445 (10%)	7,774,364 (9%)	2,953,942 (13%)	
Former	16,365,668 (15%)	11,229,636 (13%)	5,226,206 (23%)	
Mild	40,368,647 (37%)	31,961,272 (37%)	8,180,148 (36%)	
Moderate	17,456,712 (16%)	14,684,909 (17%)	3,408,395 (15%)	
Heavy	24,002,979 (22%)	20,731,636 (24%)	2,953,942 (13%)	
Hypertension (%)				< 0.001
No	67,644,759 (62%)	57,011,999 (66%)	8,861,827 (39%)	
Yes	41,459,691 (38%)	29,369,818 (34%)	13,860,806 (61%)	
Diabetes (%)				< 0.001
No	93,829,827 (86%)	76,879,817 (89%)	16,587,522 (73%)	
Yes	15,274,623 (14%)	9,501,999 (11%)	6,135,111 (27%)	
Hyperlipidemia (%)				< 0.001
No	32,731,335 (30%)	26,778,363 (31%)	4,771,753 (21%)	
Yes	76,373,115 (70%)	59,603,453 (69%)	17,950,880 (79%)	
Energy (kcal) (mean [SE])	2160.36 (989.09)	2191.43 (994.31)	2011.12 (937.81)	< 0.001
Protein (g) (mean [SE])	83.03 (42.63)	84.47 (43.15)	75.81 (39.02)	< 0.001
Carbohydrate (g) (mean [SE])	255.48 (125.55)	258.05 (126.48)	242.61 (119.88)	< 0.001
Total sugars (g) (mean [SE])	113.39 (78.11)	113.96 (78.37)	110.82 (76.61)	0.015
Dietary fiber (g) (mean [SE])	16.86 (10.28)	16.78 (10.42)	16.12 (10.24)	< 0.001
Total fat (g) (mean [SE])	83.76 (47.01)	85.11 (47.28)	77.68 (44.79)	< 0.001
DKR, Quartile (%)				0.013
Q1	27,276,113 (25%)	21,595,451 (25%)	6,135,110 (27%)	
Q2	27,276,147 (25%)	21,595,642 (25%)	5,680,658 (25%)	
Q3	27,276,151 (25%)	21,595,322 (25%)	5,453,432 (24%)	
Q4	27,276,132 (25%)	21,595,554 (25%)	5,453,421 (24%)	
DII (mean [SE])	1.28 (1.85)	1.23 (1.84)	1.49 (1.85)	< 0.001
WWI (mean [SE])	9.98 (0.85)	9.12 (1.37)	11.28 (0.19)	< 0.001

*Note:* Mean (SE) for continuous variables: the *p* value was calculated by the weighted *t*‐test. Percentages (95% CI) for categorical variables: the *p* value was calculated by the weighted Chi‐squared test.

Abbreviations: DII, dietary inflammatory index; DKR, dietary ketogenic ratio; OAB, overactive bladder; PIR, poverty income ratio; WWI, Weight‐adjusted‐waist index.

Regarding lifestyle factors, smoking status and alcohol consumption patterns differed significantly between groups (*p* < 0.001), with a higher prevalence of OAB observed among former smokers and non‐drinkers. Participants with hypertension, diabetes, and hyperlipidemia exhibited a substantially higher prevalence of OAB compared with those without these conditions (all *p* < 0.001).

In terms of dietary intake, participants with OAB had significantly lower mean intakes of total energy, protein, carbohydrates, dietary fiber, and total fat compared with those without OAB (all *p* < 0.001), while total sugar intake also differed between groups (*p* = 0.015). Moreover, the OAB group showed lower dietary ketogenic ratio (DKR) levels (*p* = 0.013), higher Dietary Inflammatory Index (DII) scores (*p* < 0.001), and higher weight‐adjusted waist index (WWI) values (*p* < 0.001).

### 3.2 Relationship Between OAB, DKR, DII, and WWI

**FIGURE 2:**
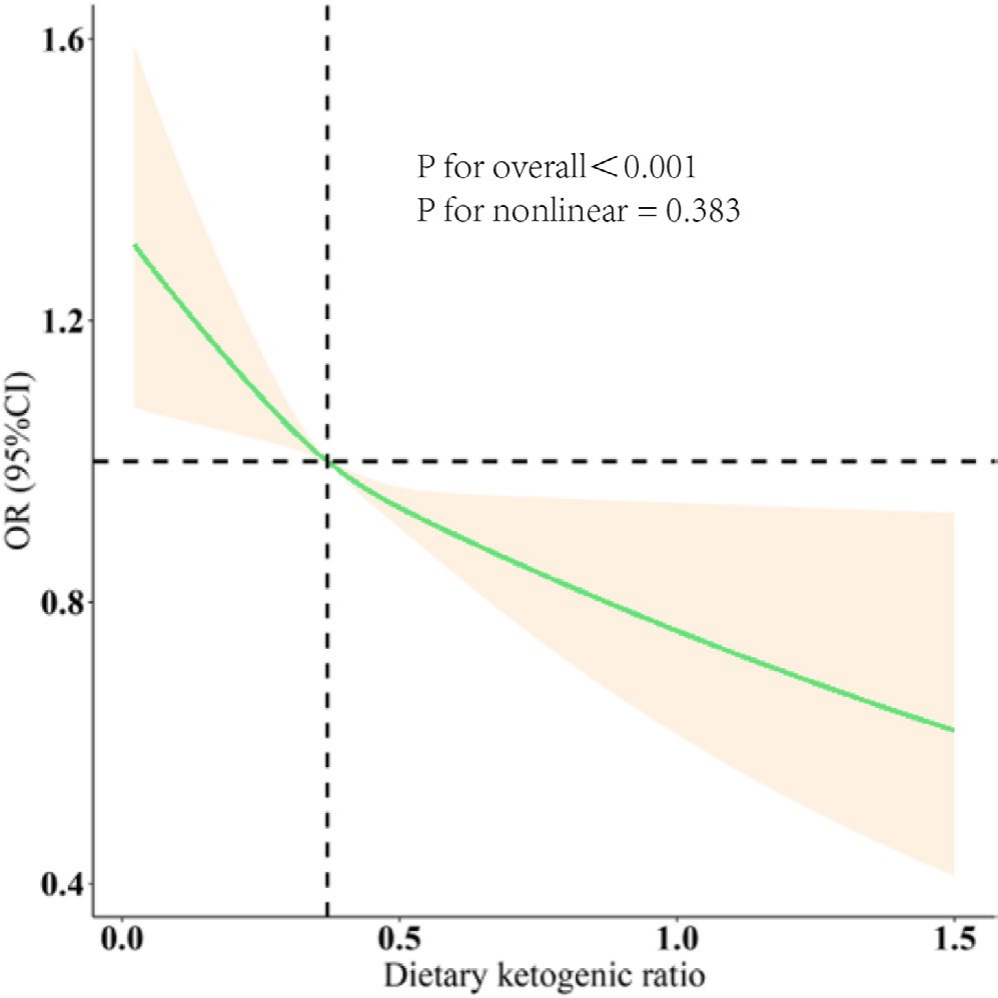
Association between the dietary ketogenic ratio (DKR) and the incidence of OAB.

**FIGURE 3:**
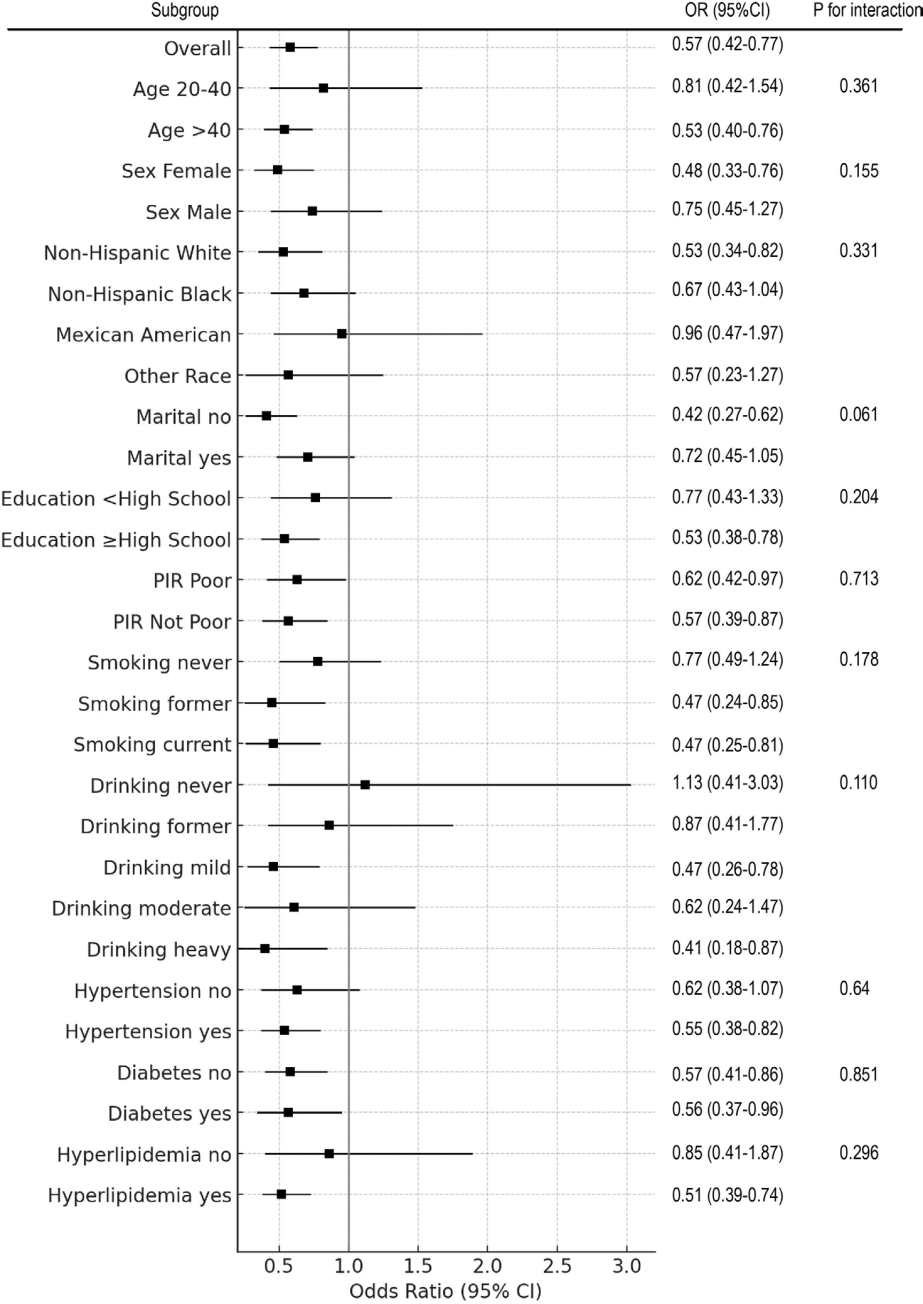
Subgroup analyses the affiliation between the dietary ketogenic ratio (DKR) and the incidence of Overactive Bladder (OAB).

The Relationship between DKR and OAB was examined using three progressively modified models (Table 2). Within the completely altered model (Model 3), each standard deviation increase in DKR was associated with a 41% reduction in the odds of having OAB [OR = 0.59, 95% CI: 0.44–0.79]. Additionally, participants in the highest DKR quartile (Q4) had a 14% lower likelihood of OAB compared to those in the lowest quartile (Q1) [OR = 0.86, 95% CI: 0.77–0.96]. A significant dose–response trend was observed across quartiles (p for trend = 0.028). Results from Models 1 and 2 were consistent with these findings. Figure 2 illustrates a significant inverse association between DKR and OAB prevalence (overall *p* < 0.001; *p* for nonlinearity = 0.383). Subgroup analyses further revealed that DKR was positively associated with OAB risk across most stratified subgroups (Figure 3).

**TABLE 2:** The relationship between DKR and OAB under multiple groups and multiple models.

Characteristics	Model 1 [OR (95% CI)]	*p*	Model 2 [OR (95% CI)]	*p*	Model 3 [OR (95% CI)]	*p*
DKR–OAB						
Continuous	0.57 (0.44, 0.75)	< 0.001	0.64 (0.49, 0.85)	0.001	0.59 (0.44, 0.79)	< 0.001
Quartile						
Q1	1 (ref.)		1 (ref.)		1 (ref.)	
Q2	0.88 (0.82, 0.99)	0.021	0.95 (0.84, 1.05)	0.242	0.92 (0.81, 1.04)	0.121
Q3	0.87 (0.77, 0.98)	0.012	0.94 (0.82, 1.07)	0.236	0.89 (0.77, 1.01)	0.066
Q4	0.83 (0.76, 0.95)	0.002	0.90 (0.80, 1.05)	0.118	0.86 (0.77, 0.96)	0.029
*p* for trend	0.004		0.126		0.028	

*Note:* Model 1: No covariates were adjusted. Model 2: age, gender, education level, marital status, PIR, and race were adjusted. Model 3: age, gender, education level, marital status, PIR, race, smoking, drinking, hypertension, diabetes, and hyperlipidemia were adjusted.

Abbreviations: CI, confidence interval; DKR, dietary ketogenic ratio; OAB, overactive bladder; OR, odds ratio.

**FIGURE 4:**
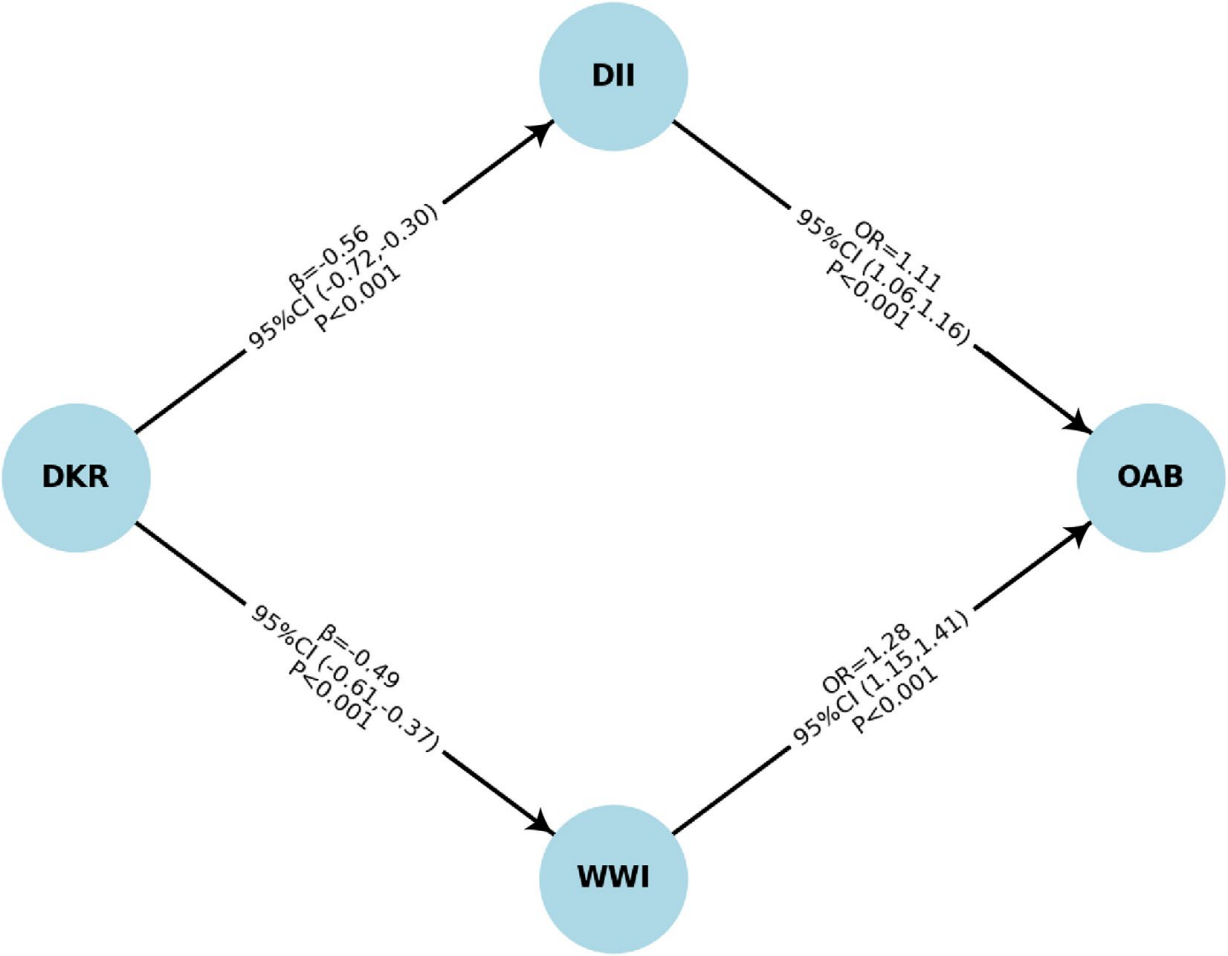
Path diagram illustrating the associations of the dietary ketogenic ratio (DKR) with overactive bladder (OAB) through the dietary inflammatory index (DII) and weight‐adjusted‐waist index (WWI).

In the fully adjusted model, DII remained significantly associated with increased OAB risk [OR = 1.11, 95% CI: 1.06–1.16, *p* < 0.001] (Figure 4). Additionally, DKR was significantly inversely correlated with DII (*β* = −0.56, 95% CI: −0.72 to −0.30, *p* < 0.001). WWI was still substantially linked to a higher incidence of OAB [OR = 1.28, 95% CI: 1.15–1.41, *p* < 0.001]. DKR was significantly inversely correlated with DII (*β* = −0.49, *p* < 0.001).

### 3.3 Analysis of the Mediation Effects of WWI and DII

**FIGURE 5:**
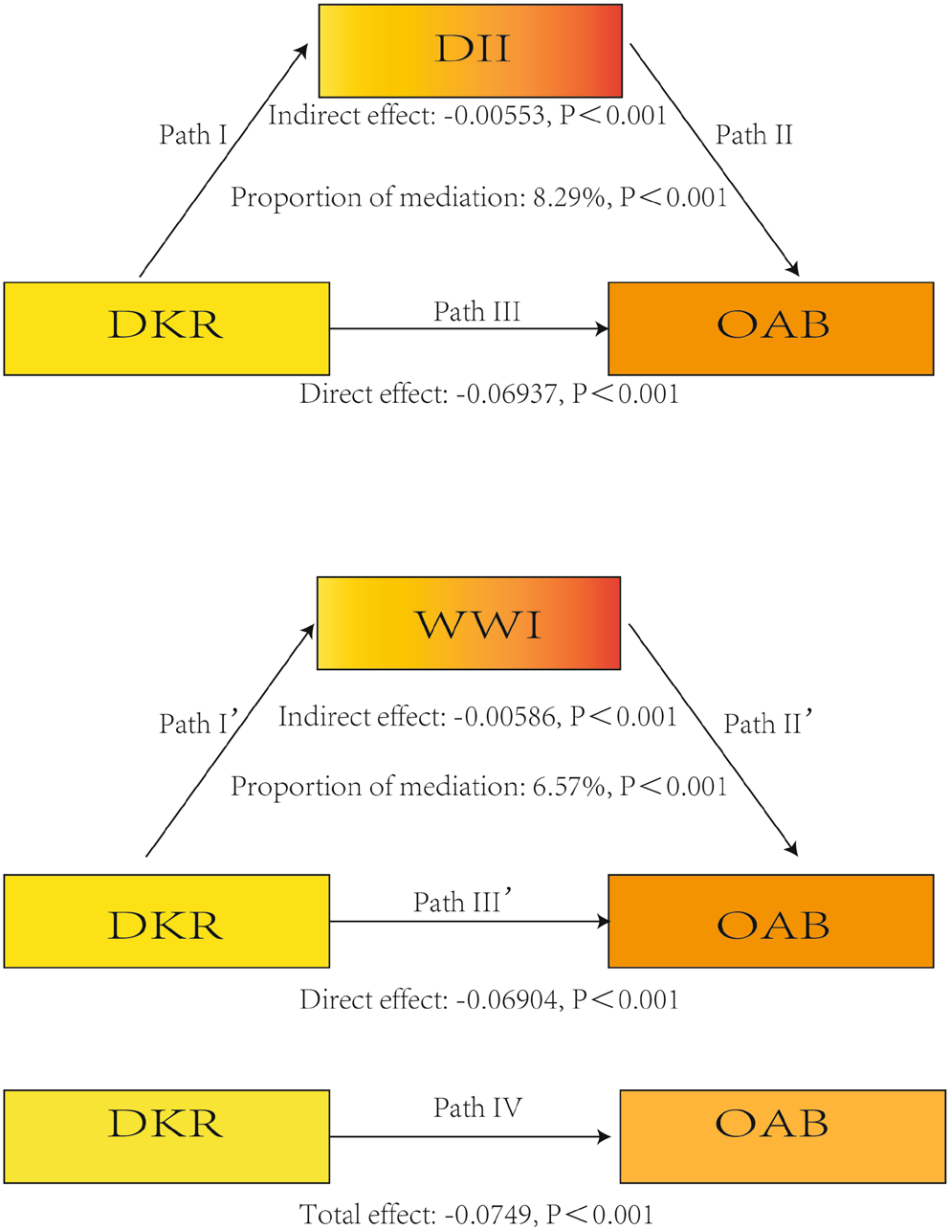
Mediation models showing the direct and indirect effects of the dietary ketogenic ratio (DKR) on overactive bladder (OAB) via the dietary inflammatory index (DII) and weight‐adjusted waist index (WWI).

Given that the exposure (DKR) was significantly associated with OAB, that DKR was significantly associated with both WWI and DII, and that WWI and DII were each significantly associated with OAB after adjustment for all covariates, the criteria for mediation analysis were satisfied (Table 3). After adjusting for all covariates, the indirect effect of WWI and DII on the association between DKR and OAB was statistically significant (Figure 5). DII mediated 8.29% of the total effect of DKR on OAB risk, with a direct effect of −6.937 × 10^−2^ (*p* < 0.001) and an indirect effect of −5.53 × 10^−3^ (*p* < 0.001). WWI mediated 6.57% of the total effect of DKR on OAB risk, with a direct effect of −6.904 × 10^−2^ (*p* < 0.001) and an indirect effect of −5.86 × 10^−3^ (*p* < 0.001). Taken together, these findings indicate that both DII and WWI act as partial mediators, suggesting that dietary patterns with higher ketogenic potential may influence OAB not only through direct biological pathways but also indirectly by modulating inflammatory status and obesity‐related measures.

**TABLE 3:** Association between DKR, DII, and OAB.

Characteristics	*β*/OR	95% CI	*p*
DKR–DII	−0.56	−0.72, −0.30	< 0.001
DII–OAB	1.11	1.06, 1.16	< 0.001
DKR–WWI	−0.49	−0.61, −0.37	< 0.001
WWI–OAB	1.28	1.15, 1.41	< 0.001

*Note:* Mediation pathway linking dietary ketogenic ratio (DKR), dietary inflammatory index (DII), and overactive bladder (OAB).

Abbreviations: DII, dietary inflammatory index; DKR, dietary ketogenic ratio; OAB, overactive bladder; WWI, weight‐adjusted‐waist index.

### 3.4 Sensitivity Analysis

To examine the robustness of the primary findings and address potential outcome misclassification, multiple sensitivity analyses were performed.

First, alternative thresholds for the constructed overactive bladder symptom score were applied. In addition to the primary definition (OABSS ≥ 3), both lower and higher cut‐off values were tested. Across these alternative thresholds, the inverse association between the dietary ketogenic ratio (DKR) and OAB remained consistent in direction and magnitude, indicating that the results were not sensitive to the choice of symptom score threshold.

Second, given that urgency urinary incontinence and nocturia represent distinct but related symptom domains of overactive bladder, each component was analyzed separately as an outcome. Higher DKR was consistently associated with a lower prevalence of urgency urinary incontinence and nocturia when modeled independently, suggesting that the observed association was not driven by a single symptom component.

Third, sex‐stratified analyses were conducted to account for known sex differences in OAB prevalence and symptom presentation. The inverse association between DKR and OAB persisted in both men and women, and no statistically significant interaction by sex was observed.

Taken together, these sensitivity analyses demonstrate that the primary findings were robust to variations in outcome definition, symptom specification, and sex stratification. Detailed results of analyses using alternative symptom score thresholds, symptom‐specific outcomes, and sex‐stratified models are provided in Tables S2–S4.

In female participants, the associations remained materially unchanged after excluding pregnant women. Detailed results are presented in Table S5.

## 4 Discussion

This study, based on a nationally representative sample of the U.S. population, identified significant inverse associations between DKR, WWI and DII with OAB. In fully adjusted models, OAB risk decreased by 41% for every unit rise in DKR (OR = 0.59), whereas OAB risk increased by 1.11 times for every unit increase in DII (OR = 1.11). In addition, OAB risk increased 1.11 times for every unit increase in WWI (OR = 1.28). Mediation analysis revealed that the connection between DKR and OAB was partly mediated via DII and WWI, suggesting that dietary intervention via DKR may reduce OAB risk by lowering dietary inflammatory levels.

To date, population‐based studies examining ketogenic dietary potential, quantified by macronutrient‐derived ketogenic indices, in relation to overactive bladder symptoms remain limited (Lv et al. 2025). KD, characterized by very low carbohydrate and normal protein intake, induces a distinct metabolic state that has historically been met with skepticism (Gardner et al. 2022). A prospective cohort study published in The Lancet demonstrated that excessive carbohydrate intake was linked to increased all‐cause mortality, prompting renewed interest in KD (Dehghan et al. 2017). KD is a therapeutic dietary pattern derived from extensive scientific research, possessing a solid theoretical foundation and feasibility (Wu et al. 2022). Evidence indicates that KD may reduce all‐cause mortality without increasing cardiovascular mortality. Its weight‐loss efficacy has been well established (Lu et al. 2023), and several underlying mechanisms have been proposed: (1) greater satiety arising from high protein intake and the appetite‐suppressing properties of ketone bodies; (2) suppression of lipogenesis with a concomitant increase in lipolysis; (3) a reduction in respiratory quotient, reflecting more efficient fat oxidation; and (4) stimulation of gluconeogenesis and elevated energy expenditure attributable to the thermic effect of protein.

Overweight and obesity have been linked to increased OAB symptoms (Zhang et al. 2024). Visceral adiposity is closely associated with sympathetic nervous system overactivity, which plays a critical role in bladder regulation, particularly detrusor and outlet control (de Groat et al. 2015). Sympathetic overactivity may increase detrusor excitability, contributing to OAB (Lin et al. 2023). Obesity‐induced elevated intra‐abdominal pressure may physically compress the bladder, raising intravesical pressure and impairing function, thereby increasing OAB risk (Zhang et al. 2024). Furthermore, obesity promotes inflammatory responses, including macrophage accumulation, which, through inflammatory mediators and growth factors, may impair bladder nerve and muscle function (Mikolaskova et al. 2024; Gregory and Saygin 2022). The multifactorial mechanisms underlying obesity‐related OAB suggest KD may reduce OAB risk by mitigating obesity. Given the strong physiological influence of pregnancy on urinary symptoms, we performed a female‐specific analysis excluding pregnant women, which yielded results consistent with the primary findings (Chen et al. 2022).

Pelvic ischemia caused by atherosclerosis is a widely accepted hypothesis in OAB pathogenesis (Tarcan et al. 2022). Urinary dysfunction reduces bladder perfusion and causes tissue hypoxia, leading to alterations in lipid metabolism, such as increased membrane lipid peroxidation, phospholipid remodeling, elevated adenosine release, and increased prostaglandin synthesis, directly affecting bladder physiology (Hsiao et al. 2020; Theodorakopoulou et al. 2019; Qu and Jiao 2023). Studies have shown that elevated levels of low‐density lipoprotein (LDL) in women's serum are negatively correlated with the risk of overactive bladder (OAB). A prospective clinical trial showed KD reduced total cholesterol (TOT‐C) (*p* = 0.01), LDL‐C (*p* < 0.01), and triglycerides (*p* = 0.02) in hypertensive obese postmenopausal women (Longo et al. 2010). Another study indicated that smaller LDL particles are more atherogenic, while KD increased LDL particle size and volume (Paoli et al. 2020). KD may thus reduce OAB risk by ameliorating atherosclerosis (Xu et al. 2023).

Oxidative stress, defined as an imbalance between reactive oxygen/nitrogen species production and clearance, results in cellular damage and is implicated in cardiovascular, metabolic, urinary diseases, and cancer (Jomova et al. 2023; Xu et al. 2019; Caliri et al. 2021). Oxidative stress indices are significantly higher in low HDL‐C groups and negatively correlated with HDL‐C levels (Jabarpour et al. 2024; Wang et al. 2025; Dağlı et al. 2025; Lin et al. 2025; Lim et al. 2025). OAB is closely associated with hypoxia, oxidative stress, and insufficient blood supply, as adequate perfusion is essential for bladder function (Lin et al. 2024). Ischemia/reperfusion cycles cause cumulative oxidative stress, potentially leading to lower urinary tract symptoms (LUTS), including OAB (Wu et al. 2021). Recent studies show carbohydrate restriction significantly lowers total cholesterol, increases HDL, and reduces triglycerides, suggesting KD may reduce OAB risk via oxidative stress reduction (Zheng et al. 2023).

KD may also influence cognition and mood regulation by altering gut microbiota and their metabolites, especially short‐chain fatty acids (Qin et al. 2024). Short‐chain fatty acids serve as energy for intestinal epithelial cells and exhibit anti‐inflammatory properties that enhance gut health (Yan, Ye, et al. 2023). Although traditionally considered sterile, the bladder harbors a low‐abundance microbial community, with more than half of the species overlapping with those found in the gut, suggesting that they may originate from the intestine (Schüroff et al. 2021). Neurogenic communication between the bladder and gut occurs via autonomic pathways, and unique bladder immune responses may be mediated by gut microbiota (Gomelsky et al. 2018). KD modulates gut microbiota composition and function, increasing Bacteroidetes and reducing Firmicutes and Actinobacteria in epilepsy patients, implying a potential pathway for reducing OAB risk (Olson et al. 2018). Moreover, higher DKR is negatively associated with depression severity, and depression, especially moderate to severe, is linked to increased OAB risk with age‐dependent variation (Aucoin et al. 2021). Thus, KD may reduce OAB risk partly by alleviating depressive symptoms.

Insulin resistance (IR), characterized by impaired insulin response and glucose uptake, has been implicated in OAB pathogenesis (Huang et al. 2025). Serum insulin and HOMA‐IR values were significantly higher in OAB patients (Kim et al. 2021). KD has been shown to improve insulin sensitivity, potentially lowering OAB risk. Higher scores indicate a more pro‐inflammatory effect. The Dietary Inflammatory Index (DII) measures the inflammatory potential of dietary patterns (Tian et al. 2021). Inflammation induces functional and structural changes in bladder smooth muscle, contributing to detrusor overactivity and OAB, potentially via cytokines that promote inflammation, like IL‐6 and TNF‐α (Di et al. 2022; Xie et al. 2021; Chen et al. 2021). Chronic low‐grade inflammation is a key driver of metabolic syndrome components, all implicated in OAB (Dabravolski et al. 2021). Hyperglycemia may cause bladder neuropathy, while obesity and visceral fat induce inflammatory pathways impairing bladder function (Yan, Li, et al. 2023). KD reduces obesity, visceral fat, and systemic inflammation by lowering DII, positively impacting bladder smooth muscle function (Bägli et al. 1999). Food optimization is essential in lowering the occurrence of OAB, and the significance of DII as a mediator between DKR and OAB emphasizes inflammation as a key mechanism.

Research on the connection between obesity and OAB has produced a variety of findings. While some research has found a substantial correlation between OAB and greater BMI, other studies have found no such relationship (Zhong and Wang 2025). One clinical measure used to evaluate visceral fat accumulation indirectly is waist circumference (WC) (Ross et al. 2020). The “obesity paradox” phenomenon of BMI has been presented in numerous studies, according to which individuals who are overweight or obese typically obtain superior outcomes, especially in individuals with acute myocardial infarction and coronary artery disease (Fukuoka et al. 2019). The incapacity of WC and BMI to differentiate between fat mass and muscle mass could be the cause of this surprising paradox (Kim et al. 2023). Compared to the conventional BMI‐based formula, the recently created WWI, which measures obesity, is more accurate in predicting the percentage of body fat. Recent studies have verified WWI's capacity to discriminate between muscle and fat mass, and its use has expanded to a number of domains, such as obesity and cardiovascular disease (Kim et al. 2022). The mechanisms relating to OAB and WWI are now poorly understood. There could be several systems at play. First, mechanical forces that raise intra‐abdominal and bladder pressure may be the source of a rise in WWI (Mohmand and Goldfarb 2011). Furthermore, noradrenergic sympathetic activity and urothelial irritation may result from the presence of the inflammatory cytokines and released leptin generated by visceral adipose tissue (Dursun et al. 2014). The substantial association between metabolic syndrome and abdominal fat accumulation—which is thought to be connected to persistent pelvic ischaemia and malfunction of the urothelium—has already been established by a number of studies (Peyronnet et al. 2019).

Despite the potential benefits suggested by our findings, adherence to ketogenic diets in real‐world settings can be challenging. Several studies have documented that long‐term compliance is often limited due to the restrictive nature of carbohydrate avoidance, difficulties with meal planning, gastrointestinal discomfort during the adaptive phase, and social or cultural barriers associated with high‐fat dietary patterns (English et al. 2021). Moreover, strict carbohydrate restriction may pose challenges for long‐term adherence, including dietary monotony, social and cultural constraints, gastrointestinal discomfort during the adaptation phase, and difficulty maintaining the diet in non‐controlled settings. These real‐world limitations may influence the extent to which ketogenic dietary patterns can be sustained over time and should be considered when interpreting population‐based associations derived from macronutrient ratios. This study has several strengths. First, as a non‐pharmacological intervention, dietary therapy and body fat management offer practical approaches to OAB risk reduction. Second, the use of nationally representative weighted NHANES data from 2009 to 2018 enhances generalizability and applicability. Third, mediation analysis provided mechanistic insight into the role of dietary inflammation and waist circumference‐related indicators in linking DKR and OAB, offering novel prevention strategies. Finally, comprehensive adjustment for confounders ensured robustness and reliability.

The cross‐sectional design is one of the limitations, as it prevents causal inference between DII, WWI, DKR, and OAB. Residual confounding from unmeasured lifestyle and genetic factors could not be excluded due to data limitations. Furthermore, OAB diagnosis relied primarily on self‐reported questionnaires, which can result in bias in measurement.

## 5 Conclusion

Based on the large sample from NHANES 2009–2018, this study found that increased DKR was significantly associated with a reduced risk of OAB. After comprehensive adjustment for potential confounders—including age, sex, race/ethnicity, education, income, marital status, smoking, alcohol consumption, hypertension, diabetes, and hyperlipidemia—the inverse association between DKR and OAB remained robust, exhibiting a clear dose–response relationship and statistical significance. Analysis of mediation showed that DII and WWI mediated the association between DKR and OAB, taking responsibility for 8.29% (DII) and 6.57% (WWI) of the total effect. These findings suggest that dietary modification aimed at increasing the ketogenic ratio and reducing dietary inflammatory potential may have beneficial implications for the prevention and management of OAB, providing evidence‐based dietary intervention guidance for affected populations. Moreover, this study offers novel insight into the diet–inflammation–disease pathway underlying OAB pathogenesis and has potential relevance for informing public health policy.

## Author Contributions

**Xuefeng Jin:** conceptualization, methodology, writing – original draft, writing – review and editing. **Tong Zhang, Hao Li, Jie Wang, Shiquan Xu**, and **Jingping Ge:** conceptualization, methodology, writing – review and editing. **Zizhi Li, Xiangrui Kong, Junlin Chen**, and **Xuejiao Wen:** conceptualization, methodology (equal). **Xiaoyan Liu** and **Hangxu Li:** supervision (supporting). **Wenhui Tong:** data curation, software.

## Funding

This work was supported by the National Natural Science Foundation of China (82370669, 82201964), the Natural Science Fund for Distinguished Young Scholars of Fujian Province (2023J06058) and the Fujian Provincial Health Commission (Middle‐Aged and Young Key Personnel Training Program, Grant No. 2023GGB03), the Fujian Provincial Department of Science and Technology (Major Science and Technology Innovation Project, Grant No. 2023Y9269), the Natural Science Foundation of Fujian Province (2023J01239).

## Ethics Statement

The Research Ethics Review Board of the National Centre for Health Statistics approved the research involving humans. The studies were carried out following local legislative and institutional requirements.

## Consent

The participants provided informed written consent to participate in this study.

## Conflicts of Interest

The authors declare no conflicts of interest.

## Supporting information


**Data S1:** fsn371587‐sup‐0001‐Supinfo1.csv.

**Data S2:** fsn371587‐sup‐0002‐Supinfo2.docx.

**Data S3:** fsn371587‐sup‐0003‐Supinfo3.docx.

**Table S1:** Criteria for conversion of symptom frequencies recorded in NHANES to OABSS scores.

**Table S2A:** Outcome defined as OABSS ≥ 2.
**Table S2B:** Outcome defined as OABSS ≥ 4.
**Table S3A:** Urgency.
**Table S3B:** Nocturia separately.
**Table S4:** Sex‐stratified.

**Table S5:** Multivariate analysis, women (excluding those currently pregnant, *N* = 227).

## Data Availability

The datasets analyzed in this study are publicly available from the National Health and Nutrition Examination Survey (NHANES) database (https://www.cdc.gov/nchs/nhanes/). The dataset processed by the authors is available in the 23763.csv file in the Supporting Information.
