# Supplementary material for: Association Between Ketogenic Diet and Overactive Bladder: The Mediating Roles of Dietary Inflammatory Index and Weight‐Adjusted Waist Index
Source: Food Sci Nutr. 2026 Feb 24;14(3):e71587. doi: 10.1002/fsn3.71587 (PMC12930284; doi:10.1002/fsn3.71587)
Supplement: Supplementary file 2 — Data S2: fsn371587‐sup‐0002‐Supinfo2.docx. [file FSN3-14-e71587-s002.docx]

Description of partial DII components

Due to the limited availability of all 45 original Dietary Inflammatory Index (DII) components within the NHANES dietary database, a partial DII was calculated in the present study following the standardised methodology proposed by Shivappa et al. The calculation incorporated dietary components that were consistently available across survey cycles, including total energy, carbohydrate, protein, total fat, saturated fat, monounsaturated fat, polyunsaturated fat, cholesterol, dietary fiber, vitamins A, C, and E, thiamin, riboflavin, niacin, vitamins B6 and B12, folate, iron, magnesium, zinc, selenium, and beta-carotene.

Several food parameters included in the original DII formulation were not available in NHANES and were therefore excluded from the calculation. These excluded components primarily comprised specific fatty acid subtypes (e.g., trans fat, omega-3 and omega-6 fatty acids), flavonoids and other polyphenols, and certain bioactive food constituents such as garlic, onion, tea, turmeric, ginger, and other phytochemicals. As commonly practised in NHANES-based studies, unavailable components were omitted without imputation, and the partial DII was derived by summing the inflammatory effect–weighted scores of all available dietary parameters. Higher DII values indicate a more pro-inflammatory dietary pattern, whereas lower (more negative) values reflect a more anti-inflammatory diet.
