# Supplementary material for: Association Between Ketogenic Diet and Overactive Bladder: The Mediating Roles of Dietary Inflammatory Index and Weight‐Adjusted Waist Index
Source: Food Sci Nutr. 2026 Feb 24;14(3):e71587. doi: 10.1002/fsn3.71587 (PMC12930284; doi:10.1002/fsn3.71587)
Supplement: Supplementary file 3 — Data S3: fsn371587‐sup‐0003‐Supinfo3.docx. [file FSN3-14-e71587-s006.docx]

**Supplementary Methods – Definition of lifestyle covariates**

**Smoking status (SMQ020)**

Smoking status was derived from the NHANES smoking questionnaire variable SMQ020, which asks participants whether they have smoked at least 100 cigarettes in their lifetime. Participants who answered “yes” were classified as ever smokers, whereas those who answered “no” were classified as never smokers, consistent with NHANES analytic guidelines.

**Alcohol consumption (ALQ130)**

Alcohol consumption was assessed using the NHANES alcohol use questionnaire variable ALQ130, which records the average number of alcoholic drinks consumed per day over the past 12 months. This variable was analysed according to its continuous distribution, with higher values indicating greater average daily alcohol intake.
