# Supplementary material for: Association Between Ketogenic Diet and Overactive Bladder: The Mediating Roles of Dietary Inflammatory Index and Weight‐Adjusted Waist Index
Source: Food Sci Nutr. 2026 Feb 24;14(3):e71587. doi: 10.1002/fsn3.71587 (PMC12930284; doi:10.1002/fsn3.71587)
Supplement: Supplementary file 4 — Table S1: Criteria for conversion of symptom frequencies recorded in NHANES to OABSS scores. [file FSN3-14-e71587-s003.docx]

**Table S1.** Criteria for conversion of symptom frequencies recorded in NHANES to OABSS scores.

**Abbreviations:**

NHANES, National Health and Nutrition Examination Survey; OABSS, Overactive Bladder Symptom Score.
