# Supplementary material for: Association Between Ketogenic Diet and Overactive Bladder: The Mediating Roles of Dietary Inflammatory Index and Weight‐Adjusted Waist Index
Source: Food Sci Nutr. 2026 Feb 24;14(3):e71587. doi: 10.1002/fsn3.71587 (PMC12930284; doi:10.1002/fsn3.71587)
Supplement: Supplementary file 5 — Table S2A: Outcome defined as OABSS ≥ 2. Table S2B: Outcome defined as OABSS ≥ 4. Table S3A: Urgency. Table S3B: Nocturia separately. Table S4: Sex‐stratified. [file FSN3-14-e71587-s001.docx]

S2A. Outcome defined as OABSS ≥2

| **Exposure** | **Model 1 OR (95% CI)** | **P** | **Model 2 OR (95% CI)** | **P** | **Model 3 OR (95% CI)** | **P** |
| --- | --- | --- | --- | --- | --- | --- |
| DKR (continuous, per SD) | 0.57(0.49,0.65) | <0.001 | 0.66(0.54,0.78) | <0.001 | 0.62(0.53,0.71) | <0.001 |
| Quartiles: Q1 | 1 (ref.) |  | 1 (ref.) |  | 1 (ref.) |  |
| Q2 | 0.89(0.82,0.96) | 0.023 | 0.96(0.84,1.08) | 0.247 | 0.95(0.84,1.06) | 0.125 |
| Q3 | 0.88(0.79,0.97) | 0.015 | 0.95(0.84,1.06) | 0.243 | 0.91(0.81,1.01) | 0.069 |
| Q4 | 0.85(0.76,0.94) | 0.003 | 0.95(0.84,1.06) | 0.131 | 0.87(0.84,0.90) | 0.031 |
| P for trend | 0.007 |  | 0.231 |  | 0.035 |  |

Model 1: No covariates were adjusted.

Model 2: age, gender, education level, marital status, PIR, and race were adjusted.

Model 3: age, gender, education level, marital status, PIR, race, smoking, drinking, hypertension, diabetes, and hyperlipidemia were adjusted.

Abbreviations: OAB, overactive bladder; OABSS, overactive bladder symptom score; DKR, dietary ketogenic ratio; OR, odds ratio; CI, confidence interval.

S2B. Outcome defined as OABSS ≥4

| **Characteristics** | **Model 1 [OR (95% CI)]** | ***P-value*** | **Model 2 [OR (95% CI)]** | ***P-value*** | **Model 3 [OR (95% CI)]** | ***P-value*** |
| --- | --- | --- | --- | --- | --- | --- |
| **DKR - OAB** |  |  |  |  |  |  |
| Continuous | 0.53(0.42,0.64) | <0.001 | 0.60(0.49,0.71) | 0.001 | 0.54(0.44,0.64) | <0.001 |
| Quartile |  |  |  |  |  |  |
| Q1 | 1 (ref.) |  | 1 (ref.) |  | 1 (ref.) |  |
| Q2 | 0.83(0.72,0.94) | 0.023 | 0.97(0.84,1.10) | 0.247 | 0.87(0.72,1.02) | 0.121 |
| Q3 | 0.82(0.70,0.94) | 0.014 | 0.93(0.82,1.04) | 0.239 | 0.85(0.69,1.01) | 0.065 |
| Q4 | 0.76(0.61,0.91) | 0.005 | 0.84(0.63,1.05) | 0.118 | 0.84(0.77,0.91) | 0.029 |
| *P for trend* | 0.004 |  | 0.129 |  | 0.033 |  |

Model 1: No covariates were adjusted.

Model 2: age, gender, education level, marital status, PIR, and race were adjusted.

Model 3: age, gender, education level, marital status, PIR, race, smoking, drinking, hypertension, diabetes, and hyperlipidemia were adjusted.

Abbreviations: OAB, overactive bladder; OABSS, overactive bladder symptom score; DKR, dietary ketogenic ratio; OR, odds ratio; CI, confidence interval.

Supplementary Table S3A（Urgency）

| **Characteristics** | **Urgency urinary incontinence Model 1 [OR (95% CI)]** | ***P-value*** | **Model 2 [OR (95% CI)]** | ***P-value*** | **Model 3 [OR (95% CI)]** | ***P-value*** |
| --- | --- | --- | --- | --- | --- | --- |
| **DKR - OAB** |  |  |  |  |  |  |
| Continuous | 0.55(0.44,0.66) | <0.001 | 0.61(0.49,0.73) | 0.001 | 0.62(0.44,0.80) | <0.001 |
| Quartile |  |  |  |  |  |  |
| Q1 | 1 (ref.) |  | 1 (ref.) |  | 1 (ref.) |  |
| Q2 | 0.82(0.69,0.95) | 0.024 | 0.93(0.84,1.02) | 0.242 | 0.91(0.76,1.06) | 0.124 |
| Q3 | 0.85(0.77,0.93) | 0.018 | 0.91(0.76,1.06) | 0.236 | 0.87(0.72,1.02) | 0.068 |
| Q4 | 0.85(0.76,0.94) | 0.004 | 0.85(0.65,1.05) | 0.118 | 0.84(0.77,0.91) | 0.029 |
| *P for trend* | 0.006 |  | 0.129 |  | 0.026 |  |

Model 1: No covariates were adjusted.

Model 2: age, gender, education level, marital status, PIR, and race were adjusted.

Model 3: age, gender, education level, marital status, PIR, race, smoking, drinking, hypertension, diabetes, and hyperlipidemia were adjusted.

Abbreviations: OAB, overactive bladder; OABSS, overactive bladder symptom score; DKR, dietary ketogenic ratio; OR, odds ratio; CI, confidence interval.

Supplementary Table S3B（Nocturia separately）

| **Characteristics** | **Nocturia Model 1 [OR (95% CI)]** | ***P-value*** | **Model 2 [OR (95% CI)]** | ***P-value*** | **Model 3 [OR (95% CI)]** | ***P-value*** |
| --- | --- | --- | --- | --- | --- | --- |
| **DKR - OAB** |  |  |  |  |  |  |
| Continuous | 0.55(0.44,0.66) | <0.001 | 0.62(0.57,0.67) | 0.001 | 0.56(0.44,0.68) | <0.001 |
| Quartile |  |  |  |  |  |  |
| Q1 | 1 (ref.) |  | 1 (ref.) |  | 1 (ref.) |  |
| Q2 | 0.86(0.82,0.90) | 0.023 | 0.93(0.84,1.02) | 0.242 | 0.91(0.80,1.02) | 0.121 |
| Q3 | 0.81(0.77,0.85) | 0.011 | 0.94(0.81,1.07) | 0.236 | 0.87(0.71,1.03) | 0.066 |
| Q4 | 0.78(0.76,0.80) | 0.002 | 0.87(0.73,1.01) | 0.118 | 0.86(0.77,0.95) | 0.026 |
| *P for trend* | 0.008 |  | 0.123 |  | 0.025 |  |

Model 1: No covariates were adjusted.

Model 2: age, gender, education level, marital status, PIR, and race were adjusted.

Model 3: age, gender, education level, marital status, PIR, race, smoking, drinking, hypertension, diabetes, and hyperlipidemia were adjusted.

Abbreviations: OAB, overactive bladder; OABSS, overactive bladder symptom score; DKR, dietary ketogenic ratio; OR, odds ratio; CI, confidence interval.

Supplementary Table S4A（Sex-stratified）

| **Characteristics** | **Men**  **Model 1 [OR (95% CI)]** | ***P-value*** | **Model 2 [OR (95% CI)]** | ***P-value*** | **Model 3 [OR (95% CI)]** | ***P-value*** |
| --- | --- | --- | --- | --- | --- | --- |
| **DKR - OAB** |  |  |  |  |  |  |
| Continuous | 0.55(0.44,0.66) | <0.001 | 0.62(0.53,0.71) | 0.001 | 0.56(0.44,0.68) | <0.001 |
| Quartile |  |  |  |  |  |  |
| Q1 | 1 (ref.) |  | 1 (ref.) |  | 1 (ref.) |  |
| Q2 | 0.86(0.82,0.90) | 0.020 | 0.96(0.85,1.07) | 0.243 | 0.93(0.81,1.05) | 0.124 |
| Q3 | 0.87(0.77,0.97) | 0.011 | 0.95(0.82,1.08) | 0.237 | 0.92(0.77,1.07) | 0.067 |
| Q4 | 0.81(0.76,0.86) | 0.003 | 0.91(0.80,1.02) | 0.119 | 0.85(0.77,0.93) | 0.024 |
| *P for trend* | 0.002 |  | 0.125 |  | 0.029 |  |

Model 1: No covariates were adjusted.

Model 2: age, education level, marital status, PIR, and race were adjusted.

Model 3: age, education level, marital status, PIR, race, smoking, drinking, hypertension, diabetes, and hyperlipidemia were adjusted.

Abbreviations: OAB, overactive bladder; OABSS, overactive bladder symptom score; DKR, dietary ketogenic ratio; OR, odds ratio; CI, confidence interval.

Supplementary Table S4B（Sex-stratified）

| **Characteristics** | **Women Model 1 [OR (95% CI)]** | ***P-value*** | **Model 2 [OR (95% CI)]** | ***P-value*** | **Model 3 [OR (95% CI)]** | ***P-value*** |
| --- | --- | --- | --- | --- | --- | --- |
| **DKR - OAB** |  |  |  |  |  |  |
| Continuous | 0.54(0.43,0.65) | <0.001 | 0.63(0.49,0.77) | 0.001 | 0.58(0.46,0.70) | <0.001 |
| Quartile |  |  |  |  |  |  |
| Q1 | 1 (ref.) |  | 1 (ref.) |  | 1 (ref.) |  |
| Q2 | 0.86(0.82,0.90) | 0.017 | 0.93(0.84,1.02) | 0.244 | 0.93(0.81,1.05) | 0.120 |
| Q3 | 0.84(0.77,0.91) | 0.011 | 0.92(0.81,1.03) | 0.233 | 0.87(0.73,1.01) | 0.066 |
| Q4 | 0.82(0.76,0.88) | 0.003 | 0.90(0.76,1.04) | 0.115 | 0.88(0.79,0.97) | 0.032 |
| *P for trend* | 0.006 |  | 0.123 |  | 0.029 |  |

Model 1: No covariates were adjusted.

Model 2: age, education level, marital status, PIR, and race were adjusted.

Model 3: age, education level, marital status, PIR, race, smoking, drinking, hypertension, diabetes, and hyperlipidemia were adjusted.

Abbreviations: OAB, overactive bladder; OABSS, overactive bladder symptom score; DKR, dietary ketogenic ratio; OR, odds ratio; CI, confidence interval.
