# Supplementary material for: Association Between Ketogenic Diet and Overactive Bladder: The Mediating Roles of Dietary Inflammatory Index and Weight‐Adjusted Waist Index
Source: Food Sci Nutr. 2026 Feb 24;14(3):e71587. doi: 10.1002/fsn3.71587 (PMC12930284; doi:10.1002/fsn3.71587)
Supplement: Supplementary file 6 — Table S5: Multivariate analysis, women (excluding those currently pregnant, N = 227). [file FSN3-14-e71587-s004.docx]

Supplementary Table S5 Multivariate analysis, women (excluding those currently pregnant, N=227)

| **Characteristics** | **Women Model 1 [OR (95% CI)]** | ***P-value*** | **Model 2 [OR (95% CI)]** | ***P-value*** | **Model 3 [OR (95% CI)]** | ***P-value*** |
| --- | --- | --- | --- | --- | --- | --- |
| **DKR - OAB** |  |  |  |  |  |  |
| Continuous | 0.55(0.44,0.66) | <0.001 | 0.64(0.53,0.75) | 0.001 | 0.59(0.48,0.70) | <0.001 |
| Quartile |  |  |  |  |  |  |
| Q1 | 1 (ref.) |  | 1 (ref.) |  | 1 (ref.) |  |
| Q2 | 0.87(0.82,0.92) | 0.016 | 0.93(0.83,1.03) | 0.243 | 0.94(0.81,1.07) | 0.121 |
| Q3 | 0.85(0.77,0.93) | 0.012 | 0.93(0.81,1.05) | 0.234 | 0.88(0.73,1.03) | 0.066 |
| Q4 | 0.83(0.76,0.90) | 0.002 | 0.91(0.79,1.03) | 0.116 | 0.89(0.80,0.98) | 0.033 |
| *P for trend* | 0.005 |  | 0.125 |  | 0.028 |  |

Model 1: No covariates were adjusted.

Model 2: age, education level, marital status, PIR, and race were adjusted.

Model 3: age, education level, marital status, PIR, race, smoking, drinking, hypertension, diabetes, and hyperlipidemia were adjusted.

Abbreviations: OAB, overactive bladder; OABSS, overactive bladder symptom score; DKR, dietary ketogenic ratio; OR, odds ratio; CI, confidence interval.
